# Using Large Language Models to Abstract Complex Social Determinants of Health From Original and Deidentified Medical Notes: Development and Validation Study

**DOI:** 10.2196/63445

**Published:** 2024-11-19

**Authors:** Alexandra Ralevski, Nadaa Taiyab, Michael Nossal, Lindsay Mico, Samantha Piekos, Jennifer Hadlock

**Affiliations:** 1 Institute for Systems Biology Seattle, WA United States; 2 Providence Health & Services Renton, WA United States; 3 Tegria, LLC Madison, WI United States; 4 Department of Biomedical Informatics and Medical Education University of Washington Seattle, WA United States

**Keywords:** housing instability, housing insecurity, housing, machine learning, artificial intelligence, AI, large language model, LLM, natural language processing, NLP, electronic health record, EHR, electronic medical record, EMR, social determinants of health, exposome, pregnancy, obstetric, deidentification

## Abstract

**Background:**

Social determinants of health (SDoH) such as housing insecurity are known to be intricately linked to patients’ health status. More efficient methods for abstracting structured data on SDoH can help accelerate the inclusion of exposome variables in biomedical research and support health care systems in identifying patients who could benefit from proactive outreach. Large language models (LLMs) developed from Generative Pre-trained Transformers (GPTs) have shown potential for performing complex abstraction tasks on unstructured clinical notes.

**Objective:**

Here, we assess the performance of GPTs on identifying temporal aspects of housing insecurity and compare results between both original and deidentified notes.

**Methods:**

We compared the ability of GPT-3.5 and GPT-4 to identify instances of both current and past housing instability, as well as general housing status, from 25,217 notes from 795 pregnant women. Results were compared with manual abstraction, a named entity recognition model, and regular expressions.

**Results:**

Compared with GPT-3.5 and the named entity recognition model, GPT-4 had the highest performance and had a much higher recall (0.924) than human abstractors (0.702) in identifying patients experiencing current or past housing instability, although precision was lower (0.850) compared with human abstractors (0.971). GPT-4’s precision improved slightly (0.936 original, 0.939 deidentified) on deidentified versions of the same notes, while recall dropped (0.781 original, 0.704 deidentified).

**Conclusions:**

This work demonstrates that while manual abstraction is likely to yield slightly more accurate results overall, LLMs can provide a scalable, cost-effective solution with the advantage of greater recall. This could support semiautomated abstraction, but given the potential risk for harm, human review would be essential before using results for any patient engagement or care decisions. Furthermore, recall was lower when notes were deidentified prior to LLM abstraction.

## Introduction

The overwhelming majority of patients in the United States have their data stored in electronic health records (EHRs). Information regarding a patient’s exposure to social determinants of health (SDoH), such as housing status, employment status, education, and quality of domestic life, provides relevant information that informs patient care and provides valuable avenues for intervention and treatment [1]. It has been estimated that SDoH can affect almost 50% of country-level variation in health outcomes, while clinical care impacts as little as 20% [2]. Housing data, in particular, including a patient’s recent housing status, are known to be intricately linked to their health status [3-5]. Therefore, gaining insight into a patient’s current and past living situation is essential to providing more complete and equitable care. It is also important for research, where capturing longitudinal exposome data is essential for analysis of health outcomes.

Housing stability is known to exist on a continuum, from complete stability (access to housing of reasonable quality in the absence of threats) to complete instability (no access to housing of reasonable quality) [6]. It is well known that people who are experiencing housing instability are at greater risk for other health issues, including substance use, comorbidities, and mental illness [3,7,8]. People facing housing instability are also at an increased risk for homelessness [5,9,10], which is associated with increased risk of morbidity and mortality [7]. Patients experiencing homelessness are also more likely to end up in the emergency department (ED), have longer hospital stays than low-income housed persons, and are less likely to use preventive services [3,10]. Women who are experiencing housing instability while pregnant face additional challenges, as they usually require consistent access to care throughout their pregnancy. Adverse exposures prior to and during pregnancy can put a child at an increased risk of both short- and long-term health consequences, and it is known that women who experience housing instability during pregnancy are at higher risk of adverse pregnancy outcomes, including preeclampsia, preterm birth, neonatal intensive care unit admission, and maternal morbidity [11-14].

SDoH information is rarely well documented in structured EHR data [15-17]. This leads to access barriers for health care teams and researchers. In addition, manually identifying SDoH, for example, through chart abstraction, is time-consuming, expensive, and impractical to scale. Because structured data have often been optimized for purposes other than individual care or research, free-text descriptions capture greater breadth and complexity of a patient’s social and behavioral history. Existing projects, such as the Protocol for Responding to & Assessing Patients’ Assets, Risks & Experiences [18], and emerging national interoperability plans [19] are providing paths for clinicians to better capture SDoH data in structured fields, but widespread data standards for data harmonization are still in early development [20].

Non–large language model (LLM)–based methods for extraction of SDoH information from free-text notes have relied heavily on identification of keywords or phrases, using either manual or semiautomated lexicon curation or rule-based methods [21-24]. However, most of these models are vulnerable to false positives (FP) and can capture only simplified concepts related to SDoH. In addition, previous research to identify housing instability from the EHR has focused primarily on homelessness or simplified housing-related concepts [24-26]. However, because housing instability is heterogeneous with many intersecting dimensions, classifying a patient experiencing housing instability can be more complex than some aspects of social history and exposures, such as smoking. By contrast, LLMs such as OpenAI’s Generative Pre-trained Transformer (GPT) models can handle large quantities of complex, unstructured data using only simple prompts. Research using LLMs on EHR data is still in its early stages, and most work has focused on either fine-tuning models for medical relevance [27-30], comparing model performance to identify the presence or absence of SDoH statements [31,32], or using LLMs for disease diagnosis or phenotyping [33,34]. In addition, it has not been made clear whether the quality of note text flagged as relevant by GPT is similar to that of a human abstractor, and whether or not it is likely to contain hallucinatory text. LLMs such as GPT could also perpetuate health inequity if they perform differently for different patient populations. It is therefore important to test for bias in these models to inform future decisions regarding the use of LLMs in the health care setting. In addition, the possibility of using deidentified clinical notes for abstraction is appealing for further supporting patient privacy. However, deidentification processes involve obfuscation of important details, including dates and locations. This may alter the semantic underpinnings of a given text, making it difficult for an LLM to accurately identify and label SDoH within a given note.

We examined whether LLMs were able to identify housing instability in clinical free-text notes with greater accuracy than manual abstraction, regular expressions (RegEx), and a pretrained named entity recognition model for SDoH, using EHRs for a population of pregnant women. We also examined the ability to distinguish between present and past instability, the possibility of algorithmic bias in the predictions made by GPT-4 and GPT-3.5, and LLM performance on deidentified versions of patient notes. In addition, we asked both LLMs for a justification of their housing label, helping to provide a better understanding of how these models understand and interpret data. These methods provide new insight relevant to housing security and use of LLMs for SDoH data that is valuable for retrospective research and to help identify patients who might benefit from proactive outreach.

## Methods

### Ethical Considerations

This retrospective study protocol was performed in compliance with the Health Insurance Portability and Accountability Act Privacy Rule and was approved by the institutional review board at Providence Health and Services study (2020000783). Consent was waived because disclosure of protected health information (PHI) for the study was determined to involve no more than a minimal risk to the privacy of individuals. All data remained within the secure Providence system. Manual and automated review of notes was conducted solely by the authors, using internal Providence resources.

### Study Setting and Participants

**Figure 1:**
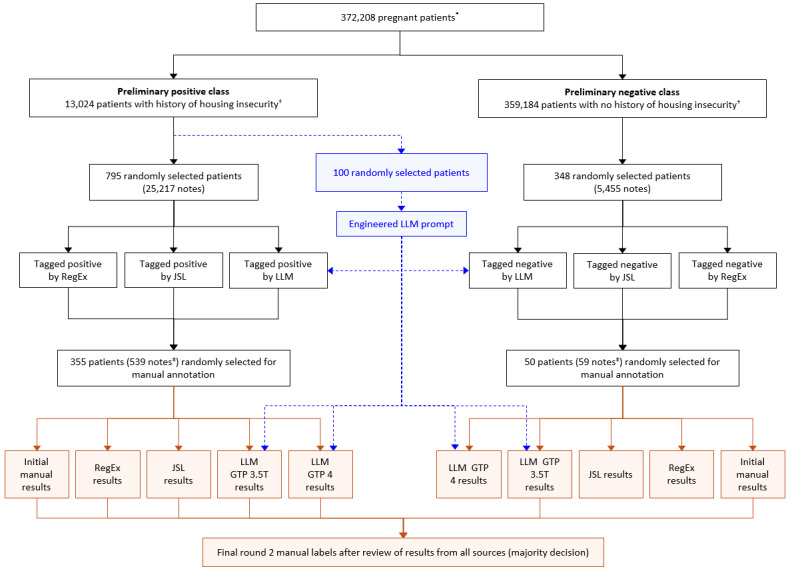
Cohort selection and experimental design. *Pregnant patients were selected as previously described. †For the positive class, charts were selected that included the SNOMED code for housing insecurity or had 1 or more notes containing the word “homeless.” ‡The most recent note tagged by each method was selected. GPT 3.5T: GPT 3.5 Turbo; JSL: John Snow Labs; LLM: large language model; RegEx: regular expressions.

Providence Health and Services is an integrated US community health care system that provides care in urban and rural settings across 7 states: Alaska, California, Montana, Oregon, New Mexico, Texas, and Washington. Using the Providence EHRs, we identified deliveries from June 8, 2010, through May 29, 2023 (N=595,600). We included singleton deliveries in a cohort of pregnant people aged 18-44 years at the start of pregnancy (n=557,406) as previously described [35]. We limited the deliveries to records that had associated gravida, term, preterm, abortion, and living data information, and to patients who received care in the Providence system during pregnancy. For patients with more than 1 pregnancy episode, we randomly selected a single episode (n=408,158). We limited our patient cohort to those with complete Social Vulnerability Index (n=372,208) information as previously described [35]. To identify patients from our cohort who were experiencing housing instability (“preliminary positive class”), we searched for patients who had either a Systematized Nomenclature of Medicine Clinical Terminology (SNOMED-CT) code for housing instability (Table S1 in Multimedia Appendix 1) or a matching string for the word “homeless” in 1 or more of their free-text notes (n=13,024). Patients who did not meet these criteria were considered in the “preliminary negative class” (n=359,184; Figure 1 [35]).

### Models

Data processing was accomplished using the Azure AI Services Application Programming Interface for GPT-4 and GPT-3.5 within the secure Providence cloud environment. GPT-4 version 0613 had a 32K token window [36], while GPT-3.5 Turbo (hereafter, GPT-3.5) version 0613 had a 16K token window [37]. Both GPT models were run using LangChain and OpenAI libraries. The John Snow Labs (JSL) named entity recognition model (ner_sdoh_en_4.4.3_3.0_1686654976160) [36] is a state-of-the-art SDoH model designed to detect and label SDoH entities within text data. The housing-specific label includes entities related to the conditions of the patient’s living spaces, for example, homeless, housing, small apartment, and so forth. JSL was run using sparknlp_jsl.version 5.0.0. To determine whether a RegEx search would identify relevant patient notes related to housing, we generated a preliminary list of keywords and phrases related to housing instability. The final list was reviewed by a registered nurse and clinical informaticist with 3 decades of experience (Table S2 in Multimedia Appendix 1).

### Prompt Engineering

Our prompt was developed using chain-of-thought prompting, where the problem or question description is initially stated and the LLM is asked to identify relevant evidence first and then provide an answer. This method has been shown to be more accurate than asking the LLM to provide only an answer [37]. GPT-4 and GPT-3.5 were asked to first identify chunks of evidence verbatim from the text (*evidence*). The model was then asked to go through each of the 4 labels: *housing noted*, *housing instability current*, *housing stability current*, and *housing instability history* and provide an answer for each. The model was then asked to provide a *justification* explaining why it chose a specific label and the LLMs were explicitly asked not to make up any information. GPT-3.5 was not used in the prompt engineering phase, and the prompt developed for GPT-4 was also used for GPT-3.5. The final prompt can be found in Multimedia Appendix 1.

### Task Definition and Data Labeling

We defined different instances of housing stability and instability by first carrying out interviews with various subject matter experts, including clinicians, social workers, and resource specialists. All abstraction was conducted by researchers AR, NT, and MN. We generated preliminary abstraction guidelines that were then iteratively refined and finalized with additional input from subject matter experts. The final abstraction guidelines can be found in Multimedia Appendix 1. These guidelines distinguish between stable and unstable housing versus an *unknown* housing status, with examples for each pulled from EHR notes. An explicit definition of *history of housing instability* was also created. If a note contained any information on housing it had to be labeled as *stably housed*, *current housing instability*, or *history of/past housing instability*. If the note contained no information on housing, it was labeled as *unknown*. These guidelines were then used to create the prompt used by the LLM. The abstraction was divided into 2 rounds, with each reviewer abstracting their own set of notes for the first round (*original label*). For the second round, 25% of the notes underwent dual abstraction. If there was any disagreement for a given note between 2 abstractors, a third abstractor was asked to make a final decision. All the notes were then manually compared with results from GPT-4 to identify any notes that were obviously missed by reviewers. After the second round, a *final* label was assigned to each note. Performance metrics for manual abstraction were calculated by comparing the *original* and *final* labels for each note. After manual abstraction from reviewers, interrater reliability was calculated using Cohen k on the notes that underwent dual abstraction during the second round of abstraction. The average time spent manually abstracting a single note was calculated by each reviewer timing himself or herself for the time spent to abstract 10 notes and then taking the average. The 2 averages for each reviewer were then averaged.

For prompt engineering, we randomly selected 100 patients from the preliminary positive class and extracted all patient notes the year prior to the patient’s conception date. Of the 100 patients, 51 had at least 1 note within the year prior to conception date, for a total of 1569 notes. Of those 51 patients, we used the JSL model to identify 70 notes from 16 patients related to housing. Each of the 70 notes were then manually abstracted by 2 different abstractors and the answers were compared. Any disagreement between the abstractors was discussed and a final decision was made. From our abstraction guidelines we developed an initial prompt for GPT-4 that contained definitions of housing instability as stated in the abstraction guidelines, as well as examples of housing instability from samples found in the patient notes. This prompt was then run through GPT-4 on the 70 notes and the results were compared with the manually abstracted results. All of the results were then compared, and the prompt was updated again based on results from GPT-4. For example, GPT-4 initially misclassified several cases of past housing instability as *current housing instability*. We then updated the prompt to specify that “a patient can only experience a ‘history’ of housing instability if they had housing instability in the past, then were stably housed, then experienced housing instability again. If the note refers to past housing instability, for example, ‘the patient was homeless in the past,’ then this can be treated as a ‘history of housing instability.’”

### Patient and Note Selection for Abstraction

Overall, the 4 methods flagged 25,217 notes from 795 patients from the preliminary positive class as being related to housing or housing instability past or present. If a given method flagged more than 1 note for a specific patient, only the most recent note in relation to the conception date was used for abstraction. For example, if GPT-4 flagged 2 notes dated January 1, 2019, and May 1, 2019, reviewers abstracted only the note dated May 1, 2019. This ensured that a maximum of 4 notes per patient were used for abstraction. Model performance was measured by examining accuracy, recall, precision, and *F*_1_-score using the *SciKit-Learn* library.

Because it was uncertain how many notes would be flagged by the models for a given number of patients, we initially selected 500 patients from the preliminary positive class. Out of those 500 patients, 295 patients had 1 or more notes within a 12-month period prior to pregnancy, for a total of 9451 notes. A full breakdown of the number of patients and notes used in each round of model tagging and abstraction can be found in Table 1 and Table S3 in Multimedia Appendix 1.

**Table 1:** Breakdown of the number of notes used in each round of model tagging and abstraction from each model for the positive and negative classes.

Class and type of notes			Notes tagged by models for housing instability annotation, n				
		JSL^a^		GPT-3.5	GPT-4	RegEx^b^	Any
**Positive class: manual annotation, round 1**							
	Most recent notes	114		73	74	82	204
	Any tagged notes	300		181	284	226	511
**Positive class: manual annotation, round 2**							
	Most recent notes	169		115	119	133	335
	Any tagged notes	552		440	546	434	900
**Negative class**							
	Most recent notes	27		11	12	14	59
	Any tagged notes	33		12	15	27	79

^a^JSL: John Snow Labs.

^b^RegEx: regular expressions.

### GPT Bias Evaluation

FPR (false-positive rate) and FNR (false-negative rate) were calculated using FP, FN (false negatives), TP (true positives), and TN (true negatives), derived from the confusion matrix:

FPR = FP/FP + TN

FNR = FN/TP + FN

Moreover, 95% CIs for a population proportion were calculated using the following formula: *CI = p̂ ± z* *× SE*, where *z*=1.96 for a 95% CL. SE was calculated using *SE = √(p̂ × (1 – p̂) / n)*, where p̂ = FPR or FNR, and n = sample size.

### Deidentification of Patient Notes

Providence has an existing corpus of deidentified notes that was created using a sequence of operations performed on text data to remove PHI [38]. These operations included multiple pretrained machine learning models and RegEx. Two versions of deidentified notes were used: *complete de-id*, in which all PHI was obfuscated and all dates were shifted or masked if shifting was not possible, and *de-id except date*, in which all PHI was obfuscated but the dates were not shifted. All notes remained within the secure Providence cloud environment.

## Results

### Manual Abstraction of EHR Notes

From the 25,217 notes from the 795 patients, the 4 automated methods flagged a total of 1411 notes (Table S3 in Multimedia Appendix 1). Given how the models were designed, JSL was able to flag only those notes related to housing in general (housing noted), while RegEx could not distinguish between current and past housing instability. Both GPT-4 and GPT-3.5 were able to flag notes related to general housing status, housing instability current, and housing instability past. After the models were run on the 25,217 notes, we selected notes that were flagged as *housing noted* for JSL, *housing instability current or past* for RegEx, and *current* *housing instability* or *past* *housing instability* for GPT-4 and GPT-3.5.

The most commonly flagged note types were assessments, plan of care, obstetric triage, history and physical, consults, discharge summary, and ED notes, indicating that these types of notes are most likely to provide relevant information related to housing or housing instability (Table S4 in Multimedia Appendix 1). We selected the 539 most recent notes from 355 patients for manual abstraction. Demographic characteristics of the 355 patients can be found in Table S5 in Multimedia Appendix 1. Of the 539 manually abstracted notes, the most common type of notes were progress notes (n=216) followed by ED provider notes (n=102) and telephone encounters (n=38; Table S6 in Multimedia Appendix 1).

Of the 182 patients who were identified as having current or past housing instability, only 18% (33/182) of patients had a structured SNOMED-CT code related to housing instability in their chart (Table 2). Although the percentage of patients with structured codes for housing instability is low, it is higher than what has been previously reported, and it is well known that structured fields do not adequately capture a patient’s housing status [31,39]. These results could reflect that these notes were selected from patients flagged for housing instability, or that health care teams are more likely to ask about and document housing instability when a patient is pregnant. A total of 10% (11/109) of patients who were labeled as *stably housed* and 11% (7/64) of patients labeled as *unknown* had a SNOMED-CT code related to housing instability (Table 2). This is likely because researchers analyzed notes only within 1 year of pregnancy, while the SNOMED-CT code could have been added to a patient’s chart at any time before pregnancy.

**Table 2:** Number of patients in each housing category who either did or did not have a SNOMED-CT^a^ code related to housing instability in their chart^b^.

Housing label	Patients with a SNOMED-CT code for homelessness, n	
	Yes	No
Stably housed	11	98
Current housing instability	32	131
Past housing instability	1	18
Unknown	7	57

^a^SNOMED-CT: Systematized Nomenclature of Medicine Clinical Terms.

^b^SNOMED-CT codes used to identify housing instability can be found in Table S1 in Multimedia Appendix 1.

Two abstractors manually abstracted the 539 notes and flagged 415 as related to housing. Of those 415, a total of 164 (30.4%) were labeled as *stably housed*, 223 (41.4%) were labeled as *current housing instability*, 28 (5%) were labeled as a *history of housing instability*, and 124 (23%) were labeled *unknown* (Table 3). Moreover, 25% (103/415) of the notes underwent dual abstraction. Before adjudication, dually abstracted notes had a Cohen k coefficient of 0.589, which reflects moderate agreement [40] and highlights the ambiguity and subjectivity of abstracting complex concepts such as housing instability compared with other SDOH mentions such as employment or parental status, despite the abstractors having been trained on the same guidelines (see Supplementary Methods in Multimedia Appendix 1).

**Table 3:** Number and Percentage of manually abstracted notes by housing label.

Housing label	Notes (n=539), n (%)
Current housing instability	223 (41.4)
Stably housed	164 (30.4)
Unknown	124 (23)
History of housing instability	28 (5)

### Identification of Current and Past Housing Instability

Because housing status can change over time, it is important to be able to distinguish between current and past housing instability. This may be especially important for certain groups of patients, such as pregnant women, where housing instability during pregnancy may have different implications than prior housing instability. Tables 4 and 5 and Tables S7 and S8 in Multimedia Appendix 1 illustrate the differences in performance metrics between GPT-4, GPT-3.5, RegEx, and manual abstraction in identifying notes related to current or past housing instability, measured against final adjudicated labels. For current and past housing instability, the recall of GPT-4 was higher (0.924) than that of GPT-3.5 (0.717), RegEx (0.649), and manual abstraction (0.702). However, manual abstraction had the highest precision among the 4 methods (0.971), compared with GPT-4 (0.850), GPT-3.5 (0.759), and RegEx (0.632). The low performance of RegEx was due, in part, to acronyms related to housing, such as *supportive living services* and *recreational vehicle*, that also serve as equivalent medical shorthand for terms such as *single limb support* and *review*. This highlights the shortcomings of using a RegEx-based approach when attempting to identify a complex concept such as housing instability. For identifying current housing instability, GPT-4 still had higher recall than both GPT-3.5 and manual abstraction, but both LLMs had lower precision than manual abstraction. The recall for GPT-3.5 was higher for current housing instability alone, indicating that this model struggled to identify notes where past housing instability was mentioned, further evidenced in Table 6. This demonstrates that while LLMs can identify past or current instances of an event such as housing instability, specific models should be tested for their performance in each category individually.

**Table 4:** Comparison of recall and precision for RegEx^a^, GPT-3.5, GPT-4, and manual abstraction in identifying notes with current or past housing instability, measured on 539 manually abstracted notes.

Model	Recall	Precision
RegEx	0.649	0.632
GPT-3.5	0.717	0.759
GPT-4	0.924	0.85
Manual annotation	0.702	0.971

^a^RegEx: regular expressions.

**Table 5:** Comparison of recall and precision for GPT-3.5, GPT-4, and manual abstraction in identifying notes with current housing instability, measured on 539 manually abstracted notes.

Model	Recall	Precision
GPT-3.5	0.753	0.734
GPT-4	0.906	0.831
Manual annotation	0.718	0.933

**Table 6:** Recall and precision metrics for GPT-4 and GPT-3.5 for each housing label measured on 539 manually abstracted notes

Housing label	GPT-4		GPT-3.5	
	Recall	Precision	Recall	Precision
Stable housing	0.39	0.88	0.34	0.67
Current housing instability	0.91	0.83	0.75	0.73
Past housing instability	0.68	0.63	0.18	0.62
Unknown	0.82	0.53	0.68	0.38

The drop in recall for manual abstraction highlights the high level of ambiguity associated with abstracting this type of information, as well as the energy needed for humans to conduct manual abstraction for extended periods of time. Examples of relevant notes, along with the housing labels and justifications from GPT-4 and GPT-3.5, can be found in Table 7. There were several ambiguous cases related to housing in patient notes. For example, “Was in domestic violence. Daughters are [redacted ages] and living with parents for now.” In this example, it is not explicitly stated where the patient is staying, making her current housing status *unknown*. However, this was flagged as *current instability* by GPT-4 and *stably housed* by GPT-3.5. In another example, a patient was living with her family and their landlord was a meth dealer. However, the patient’s boyfriend was actively trying to find them a new living situation, which made the patient happy. While both abstractors agreed that the housing situation was not stable, this type of instability was not explicitly mentioned in the note prompt for the LLMs. The reviewers labeled the note as *current instability*, but the note was tagged as *stably housed* by GPT-4 and *current instability* by GPT-3.5.

**Table 7:** Examples of relevant note text and manual abstraction labels compared with GPT-4 and GPT-3.5 and relevant justification^a^.

Manual abstraction	GPT-4 label	GPT-3.5 label	Text selected from longer note by human abstractor	Justification GPT-4	Justification GPT-3.5
Current instability	Current instability	Current instability	She has been under a great deal of stress over the past year and recently became homeless. She and her boyfriend have moved to this area where she has family where she is now staying.	The patient note mentions that the patient recently became homeless, indicating current housing instability. The patient is now staying with family, but this is likely a temporary arrangement given the recent homelessness and the reference to a halfway house, which is a form of transitional housing. There is no evidence in the note to suggest a history of housing instability prior to the current situation.	The evidence shows that the patient recently became homeless and is currently facing housing instability. The patient’s history of frequent kidney stones and recent surgeries for kidney stones further suggests a lack of stable housing.
Current instability	Past instability	Current instability	She was having many stressors at that time which included being kicked out of her mother’s house and staying with ex BF.	The patient note mentions that the patient was kicked out of her mother’s house and was staying with her ex-boyfriend. This indicates a past instance of housing instability. However, the note does not provide current information about the patient’s housing situation, so it is unknown whether the patient is currently stably housed or not.	The patient note mentions that the patient was kicked out of her mother’s house and was staying with her ex-boyfriend, which indicates current housing instability. In addition, the patient’s active problem list includes depression, which can be associated with housing instability. However, there is no explicit evidence or mention of past housing instability, so it is unknown whether the patient has a history of housing instability.
Current instability	Current instability	No housing noted, current instability unknown	She said she is staying at a motel.	The patient note mentions that the patient is currently staying at a motel, which indicates a form of housing instability. There is no evidence in the note to suggest that the patient has faced housing instability in the past.	There is no explicit evidence or mention of the patient’s housing status in the note. The patient mentions staying at a motel, but it is unclear whether this is a temporary situation or a stable housing arrangement. Therefore, the current housing stability is unknown.
Current instability	Stably housed	Current instability	Pt reports she is living w/[redacted]; they are renting a house from [redacted]; he is a meth dealer. [Bf] has made arrangements to get them into a new living situation. She is very happy about this.	The patient is currently living with [redacted], in a house they are renting. There is no evidence of housing instability at the moment. The boyfriend has made arrangements for a new living situation, which the patient is happy about. There is no mention of past housing instability.	The evidence from the patient note indicates that [the patient] is currently facing housing instability. She is living in a rented house with [redacted], but the house is owned by [redacted] who is a meth dealer. In addition, the note mentions that [the patient] is in the process of moving to [redacted] and has been applying for jobs. This indicates that her current housing situation is unstable. There is no evidence of past housing instability mentioned in the note.
Unknown	Current instability	Stably housed	States lots of changes lately. Was in domestic violence. Daughters are [redacted] and living with parents for now.	The patient note mentions that the patient’s daughters are currently living with her parents, which suggests that the patient is temporarily staying with family members. This is an indication of current housing instability. There is no evidence in the note to suggest a history of housing instability.	The patient’s housing status is noted in the evidence. The patient is currently stably housed as she is living with her parents. There is no evidence of current or past housing instability.

^a^Certain sections of note text and evidence from GPT-4 and GPT-3.5 have been removed to protect patient privacy.

A minority of notes contained specific references to housing, such as “patient lives in the woods of [redacted location] with her boyfriend.” This phrase signified likely homelessness to most abstractors, but one abstractor assumed that this meant a cabin in the woods. This case was caught by GPT-4 but missed by RegEx and GPT-3.5. There were additional cases where housing instability was explicitly mentioned and was missed by 1 or more reviewers. This most often occurred in longer notes that contained a significant amount of information, and only 1-2 sentences related to housing, for example “section 8 voucher,” which refers to a US program for assisting very low-income families. This sentence was missed by GPT-3.5 and RegEx but was correctly identified by GPT-4. In addition, there were several notes that contained no information (“blank” notes). In several cases, GPT-3.5 used sentences from the prompt text as evidence and justification and flagged the note as current or past instability. This did not occur with GPT-4. This is likely because the LLM was asked to provide text evidence verbatim, and GPT-3.5 used the prompt because no relevant note text was available. However, the researchers found no instances of hallucinated evidence in any of the GPT-4 responses that were reviewed, suggesting that requiring verbatim evidence from LLMs can be a solution to hallucinated responses.

Although GPT-3.5 struggled to identify several cases of housing instability, the researchers could not identify a consistent trend in the type or content of the FP or FN notes. However, there were several cases where GPT-3.5 listed known risk factors mentioned elsewhere in the note as evidence of housing instability. For example, it noted a patient’s frequent kidney surgeries or a depression diagnosis as justification for housing instability, although there was clear mention of housing instability elsewhere in the note (Table 7). This was not the case with GPT-4, which used only direct evidence from the note text that mentioned housing-related terms as justification. Although kidney disease and depression are associated with housing instability [41-44], a human abstractor would not use this as a justification for housing instability. Having an LLM use this as justification could be a potential concern but could also be an opportunity for a different use case, where researchers ask LLMs to identify potential risk factors observed in a set of records. Those results might show bias in LLMs or highlight patterns that humans overlook. These examples also demonstrate significant differences between the 2 GPT releases and highlight the value of providing evidence and justification for every note when using LLMs. In most cases, the evidence gathered by the reviewers was similar or identical but the interpretation differed. Similarly, the evidence gathered by GPT-4 was similar to the reviewers in most cases (although this was not always the case with GPT-3.5). This indicates that, while manual abstraction is likely to yield more accurate results, GPT-4 could be used to rapidly gather relevant note text for computer-assisted manual review, helping save time without losing important or relevant patient information.

### Identification of General Housing Status

Table 8 and Table S9 in Multimedia Appendix 1 show the performance of GPT-3.5 and GPT-4 compared with JSL and manual abstraction in identifying notes where housing was mentioned. GPT-4 outperformed both GPT-3.5 and JSL across all 4 metrics but had a slightly worse precision compared with manual abstraction (0.936 compared with 0.952), although recall was higher (0.781 compared with 0.720). Interestingly, the majority of cases that were missed by GPT-4 were instances where housing was stable, for example, “she lives at home with her children” or “patient was requesting to go home.” A possible underlying cause was that the prompt had been heavily focused on identifying cases of housing instability, with little guidance provided on identifying housing status overall. Prompt engineering focused on different proportions of relevant information might yield different and more accurate results.

**Table 8:** Comparison of recall and precision for JSL^a^, GPT-3.5, GPT-4, and manual abstraction in identifying notes where housing was noted, measured on 539 manually abstracted notes.

Model	Recall	Precision
JSL	0.733	0.864
GPT-3.5	0.675	0.875
GPT-4	0.781	0.936
Manual annotation	0.72	0.952

^a^JSL: John Snow Labs.

### LLM Performance by Housing Category

Table 6 and Tables S10 and S11 in Multimedia Appendix 1 show the differences in performance for GPT-4 and GPT-3.5 in identifying notes across the different housing categories: stable housing, current housing instability, past housing instability, or unknown. GPT-3.5 performed worse than GPT-4 across all categories and had particularly low recall for notes labeled as *past instability* compared with GPT-4, which had a higher recall than precision in this category. Both GPT-4 and GPT-3.5 demonstrated poor recall for *stable housing* notes. A possible underlying cause was that the prompt focused more heavily on housing instability and did not provide training focused on stable housing. These data indicate a performance improvement for the GPT-4 release and demonstrate the effects of prompt engineering on the model outcome.

### LLM Bias Evaluation

To test for bias in the LLMs, we used the fairness criteria of separation [45] to examine the FPR and FNR between GPT-4 and GPT-3.5 across 3 different housing labels (housing noted, housing instability past or current, and housing instability current) across the age (18-30 years and 31-44 years), race (American Indian and Alaska Native, Asian, Black, Native Hawaiian and other Pacific Islander, Unknown or Declined, White, and Other), and ethnic (Hispanic or Latino, not Hispanic or Latino, and Unknown or Declined) demographic groupings. We then examined the 95% CIs of the FPR and FNR for each group (Tables S12-S17 in Multimedia Appendix 1 and Figures S1-S3 in Multimedia Appendix 1). According to the fairness criteria of separation, any difference in FPR and FNR between groups suggests potential algorithmic bias. We did observe differences in FPR and FNR between all of the groups within the 3 demographic categories. However, when we examined the overlap of the 95% CIs between groups, we found that in all cases, except in cases where the sample sizes were extremely small (n<5), there was overlap between CIs for all the groups, suggesting that the differences between groups are not significant. However, further work with a larger sample population is needed.

### Cost Breakdown of LLMs Compared with Manual Abstraction

There was a substantial difference in cost between GPT-4 and GPT-3.5, as shown in Tables 9 and 10, due to the increase in cost per 1000 tokens for notes for GPT-4 (US $0.06) compared with GPT-3.5 (US $0.003). The output cost also increased from US $0.004 per 1000 tokens for GPT-3.5 to US $0.12 per 1000 tokens for GPT-4. Interestingly, the prompt (US $1778.39 for GPT-4) cost more than the total for all the notes (US $701.72 for GPT-4), and this was the case for GPT-3.5 as well. This is because the prompt had to be included as part of each note. Because the prompt was long (1182 tokens), this increased the cost substantially. Future work comparing model performance in relation to prompt length would provide valuable insight into this trade-off.

The cost of manual abstraction varies by location, but in the United States, it can be estimated to be the minimum wage per hour for that state. As of January 2024, in the state of Washington, the minimum wage was US $16.28 per hour [46]. To analyze 25,217 notes would have cost approximately US $9442, substantially higher than either LLM.

**Table 9:** Cost analysis of GPT-4 compared with GPT-3.5 for 25,217 notes from 795 patients^a^.

Model	Costs (US $)			
	Prompt	Notes	Output	Total
GPT-4	1788.39	701.72	181.35	2671.46
GPT-3.5	89.42	35.09	8.89	133.40

^a^GPT-4 prompt: 1182 tokens × 25217 notes × 0.06 per 1000 tokens. GPT-4 notes: 11,695,270 tokens × 0.06 per 1000 tokens. GPT-4 output: 15,11,244 tokens × 0.12 per 1000 tokens. GPT-3.5 prompt: 1182 tokens × 25,217 notes × 0.003 per 1000 tokens. GPT-3.5 notes: 11,695,270 tokens × 0.003 per 1000 tokens. GPT-3.5 output: 22,23,674 tokens × 0.004 per 1000 tokens.

**Table 10:** Estimated cost of running a single note on GPT-4 and GPT-3.5 compared with manual abstraction.

Model	Cost (US $)
Manual annotation	0.374
GPT-4	0.106
GPT-3.5	0.005

### Analysis on Patients in the Preliminary Negative Class

To evaluate the performance of our 4 methods compared with patients from the preliminary positive class, we selected a random sample of 5455 notes from 348 patients in the preliminary negative class. GPT-4, GPT-3.5, RegEx, and JSL were run on all 5455 notes using the same method as notes from patients in the preliminary positive class. Of the 5455 notes, all 4 methods flagged only 59 of the most recent notes from 50 patients with 1 of the 4 housing labels (GPT-4: n=12, GPT-3.5: n=11, RegEx: n=14, and JSL: n=27). A randomly selected sample of 20 notes from the preliminary negative class that were not flagged by any of the 4 methods was reviewed by a manual abstractor (AR). Of the 20 notes, 19 notes did not contain any information on housing and 1 note suggested stable housing. An additional randomly selected sample of 20 notes was taken from the 59 notes that were flagged by the 4 methods and reviewed by a manual abstractor (AR). Of the 20 notes, 8 were *current housing instability*, 1 was *past housing instability*, 5 were *stably housed*, and 6 were *unknown*.

This demonstrates that, as expected, patients from the preliminary negative class had far fewer notes related to housing or housing instability than patients in the preliminary positive class. To generate a manually abstracted data set of ~500 notes to compare with the preliminary positive class, the 4 methods would need to analyze approximately 46,000 notes. However, JSL is not able to distinguish between notes related to general housing and housing instability, and RegEx has low precision and recall for identifying notes related to housing instability. Therefore, LLMs provide the best chance of finding relevant notes related to housing or housing instability for patients in either class. Because the number of notes flagged by GPT-4 and GPT-3.5 were very low in the preliminary negative class (12 and 11, respectively, out of 5455 notes), the 2 models would need to analyze approximately 227,300 notes to find ~500 notes related to housing or housing instability in the preliminary negative class. Due to the cost restrictions of running these LLMs, we were unable to perform this analysis. However, future work to analyze additional notes from patients in the preliminary negative class could provide insight into any differences in notes between these 2 classes.

### Evaluation of GPT-4 on Deidentified Patient Notes

Deidentification can help mitigate privacy risks to individuals to support secondary use of data for research. In the United States, the Health Insurance Portability and Accountability Act specifies 18 categories of information that are PHI that must be removed from medical records [47,48]. However, while the process of deidentification is necessary to protect patient privacy, the information that is removed during this process, such as dates and locations, may result in the loss of important contextual clues needed for LLM analysis of housing instability. We wanted to examine whether LLMs performed similarly on two versions of deidentified patient notes compared with original notes: (1) fully deidentified notes where all PHI was obfuscated and all dates were shifted or masked if shifting was not possible (hereafter referred to as *complete de-id*), and (2) patient notes where all PHI was obfuscated but the dates were not shifted (hereafter referred to as *de-id except date*). All notes remained within the secure Providence system. All data processing was conducted in Providence’s secure cloud environment.

We ran the 2 deidentified versions of the 539 manually abstracted notes through GPT-4 and compared the performance metrics with the original notes to identify current or past housing instability or general housing status (housing noted). We found that in all cases, recall dropped but precision increased for the deidentified notes compared with the original notes (Multimedia Appendix 2 and Table S18 in Multimedia Appendix 1). For example, for the notes labeled as *current housing instability*, the recall for GPT-4 on the original notes was 0.906, but this dropped to 0.812 and 0.834 for the *complete de-id* and *de-id except date* notes, respectively. By contrast, the precision increased from 0.831 for the original notes compared with 0.862 and 0.849 for the *complete de-id* and *de-id except date* notes, respectively. These minor increases in precision are likely because results from GPT-4 are slightly different each time the model is run, resulting in slight differences in performance. The drop in recall is not surprising given the nature of deidentification, in which both places and locations have been obfuscated, making it more difficult for the model to identify relevant notes. For example, there were several cases in the original notes where the patient was stated to be living in a specific location, such as a city or county, but these locations were changed to medical facilities or, in one instance, a jail, resulting in the model sometimes mislabeling the patient as *unstably housed* or missing the note as related to housing altogether. In other cases, when the dates were shifted, instances of *past housing instability* were made current, making it difficult for the LLM to properly identify and label these notes.

## Discussion

### Principal Findings

Our results demonstrate the potential benefit of using LLMs to identify instances of complex SDoH concepts in the EHR, such as past or current housing instability. Although manual abstraction correctly classified more notes related to housing instability, it is more expensive than using LLMs, with a limited increase in performance. GPT-4 outperformed GPT-3.5, JSL, and RegEx in identifying patients experiencing current or past housing instability. In most cases, the evidence from GPT-4 was similar or identical to that of the manual abstractors, and no hallucinations were observed in GPT-4 output. Our work also suggests that requiring the LLM to provide verbatim evidence and justification from the original text can help reduce the risk that relevant context about housing information is omitted from LLM results. We also found that LLMs perform well in identifying notes related to housing insecurity without the need for fine-tuning, using only few-shot learning. However, given the sensitive nature of housing insecurity and complexity of individual situations, final human review would be essential for any decision regarding patient communication or patient care. Another important finding is that recall was lower on notes after they had been deidentified. Further research is needed to determine whether human annotators would also have lower performance on deidentified notes.

It is important to note that housing instability does not exist in a vacuum; oftentimes, there are multiple compounding factors that can either contribute to or be influenced by housing instability, including domestic violence, drug abuse, and mental illness. One limitation of this study was our focus solely on housing instability and not an additional identification of these risk factors. This resulted in some cases where a patient was technically considered to have stable housing, but there were other risk factors in the patient note that would likely be important for users of abstraction results: case workers, clinicians, or researchers. Expanding the prompt might improve performance and enable labeling that separates out multiple dimensions of housing security, including uncertainty about future housing, frequency of housing transitions, and risks from unsafe housing situations. Because GPT-4 and GPT-3.5 are not deterministic models, responses, and therefore performance, may also change if rerun on the same notes. However, the newest release of GPT, GPT-4 Turbo, allows researchers to add a deterministic seed parameter to ensure that the model returns the same response every time, helping to prevent changes in performance across multiple runs.

Because all our methods required that each note be analyzed individually, all 4 methods identified 1411 notes out of 25,217 related to housing or housing instability in the preliminary positive class, and only 64 out of 5455 notes related to housing or housing instability in the preliminary negative class, we can conclude that many notes in this study likely did not contain information on housing or housing instability. However, future work could investigate the similarities and differences in note content related to housing or housing instability between patients in both classes. In addition, because we used automated methods for the initial selection of relevant patient notes, we likely missed some patient notes related to housing or housing instability that were not captured with any of the 4 automated methods. Future work to manually abstract a larger corpus of patient notes related to housing and housing instability, as well as other SDoH categories, would be valuable for full validation. Furthermore, repeat validation over time would be important to reduce the risk of drift, and new tests would be appropriate before deploying the system in different geographic settings. . Another limitation is that the study was limited to the content documented in EHR notes, and a recent survey reported that only about 60% of patients felt comfortable sharing SDoH-related information [49]. Future studies would benefit from longitudinal confidential surveys or interviews with patients and health care teams. In addition, because the time and cost to run GPT-4 and GPT-3.5 might not be feasible across millions of patient notes, work with newly emerging open source language models may provide a similar performance for a much lower cost and run time.

### Conclusions

Methods for abstracting SDoH data can be valuable for retrospective research, chart review for prospective trials, and population health interventions to identify those who might benefit from proactive outreach. This work demonstrates that LLMs have potential for computer-assisted abstraction of social history, improving recall and reducing costs. This includes temporal feature engineering, such as identifying whether patient experienced housing instability before or during their pregnancy. At the same time, precision was slightly lower than that of human abstractors, and given the potential risk for harm to patients, human review would be essential for any decisions on patient communication or patient care. Results also identified 2 important areas where further work is needed: separating out 3 different dimensions of housing insecurity and advancing deidentification methods that do not result in loss of information on social history.

## Appendix

Multimedia Appendix 1 Supplementary methods, tables, and figures.

Multimedia Appendix 2 Comparison of GPT-4 performance in identifying (A) current or past housing instability, (B) current housing instability, or (C) general housing status from 3 different versions of the same set of 539 notes. Note versions were original patient notes, completely deidentified notes (complete de-id), or deidentified notes dates unchanged (de-id except date).

## Abbreviations

ED: emergency department
EHR: electronic health record
FN: false positive
FNR: false-negative rate
FP: false negative
FPR: false-positive rate
GPT: Generative Pre-trained Transformer
JSL: John Snow Labs
LLM: large language model
PHI: protected health information
RegEx: regular expressions
SDoH: social determinants of health
SNOMED-CT: Systematized Nomenclature of Medicine Clinical Terminology
TN: true negatives
TP: true positives

## Data Availability

Results have been aggregated and reported within this paper to the extent possible while maintaining privacy from personal health information as required by US law. All data are archived within Providence systems in a Health Insurance Portability and Accountability Act–secure audited compute environment, and those wishing to verify study conclusions can contact the chief data officer. All biomedical codes used to extract data from electronic health records have been shared. The source code used to run models and reproduce results shown for this study is available at GitHub [52].
