# Supplementary figures and images for: Using Large Language Models to Abstract Complex Social Determinants of Health From Original and Deidentified Medical Notes: Development and Validation Study
Source: J Med Internet Res. 2024 Nov 19;26:e63445. doi: 10.2196/63445 (PMC11615547; doi:10.2196/63445)

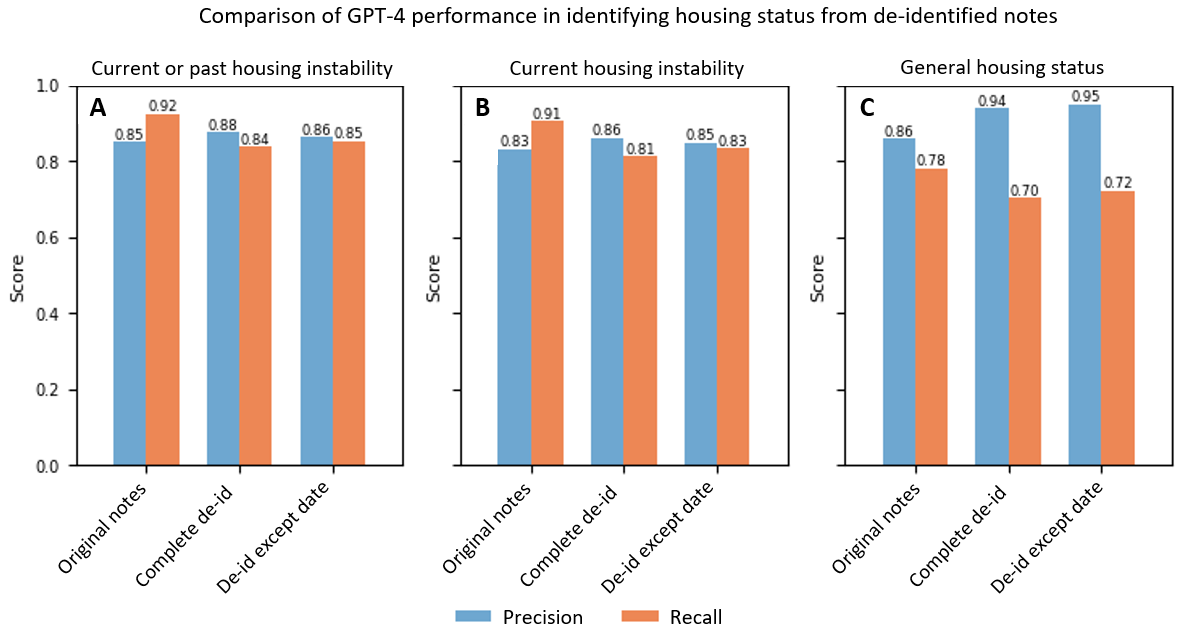

Supplement: Multimedia Appendix 2 [file jmir_v26i1e63445_app2.png]
